# Supplementary material for: Quantifying and comparing radiation damage in the Protein Data Bank
Source: Nat Commun. 2022 Mar 14;13:1314. doi: 10.1038/s41467-022-28934-0 (PMC8921271; doi:10.1038/s41467-022-28934-0)
Supplement: Supplementary file 1 — Supplementary Information [file 41467_2022_28934_MOESM1_ESM.pdf]

Supplementary Information

Quantifying and comparing radiation damage in the Protein Data Bank

Kathryn L. Shelley[1,2]* and Elspeth F. Garman[1]*

[1]Department of Biochemistry, University of Oxford, South Parks Road, Oxford, OX1 3QU, United Kingdom
[2]School of Chemistry, University of Bristol, Cantock's Close, Bristol, BS8 1TS, United Kingdom

kathryn.l.shelley@gmail.com
elspeth.garman@bioch.ox.ac.uk

Supplementary Figures

Supplementary Figure 1: Comparison of the distribution of the B_{Damage} values of all atoms in low and high dose models.

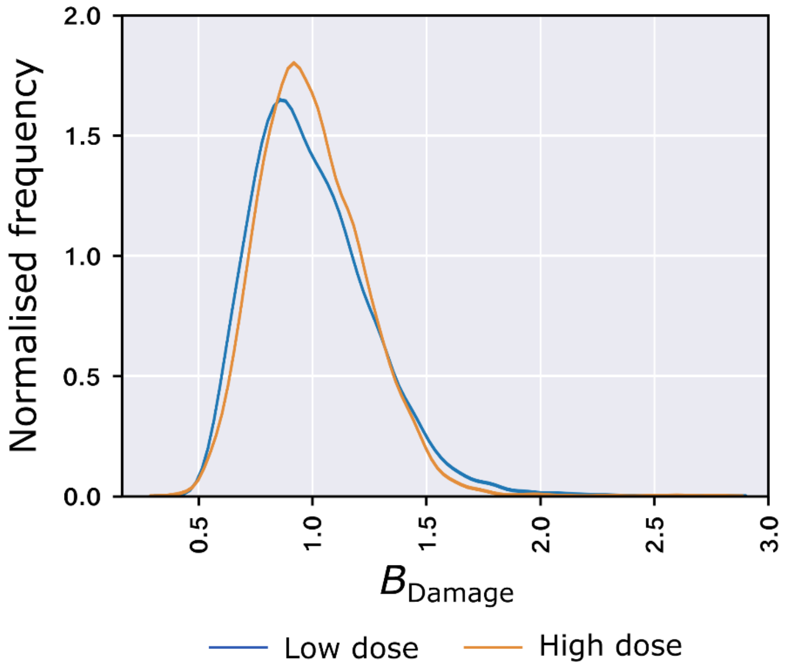

There is minimal difference between the distribution of the B_{Damage} values of all atoms in a structure in a low dose (blue curve) structure as compared to a high dose (orange curve) structure. Displayed kernel density estimates are of a low (1.11 MGy, PDB accession code 5MCC, blue curve) and a high (22.7 MGy, PDB accession code 5MCN, orange curve) dose structure of GH7 family cellobiohydrolase[1]. Source data are provided as a Source Data file.

Supplementary Figure 2: Investigating the relationship between B_{Damage} and dose for the GH7 cellobiohydrolase damage series.

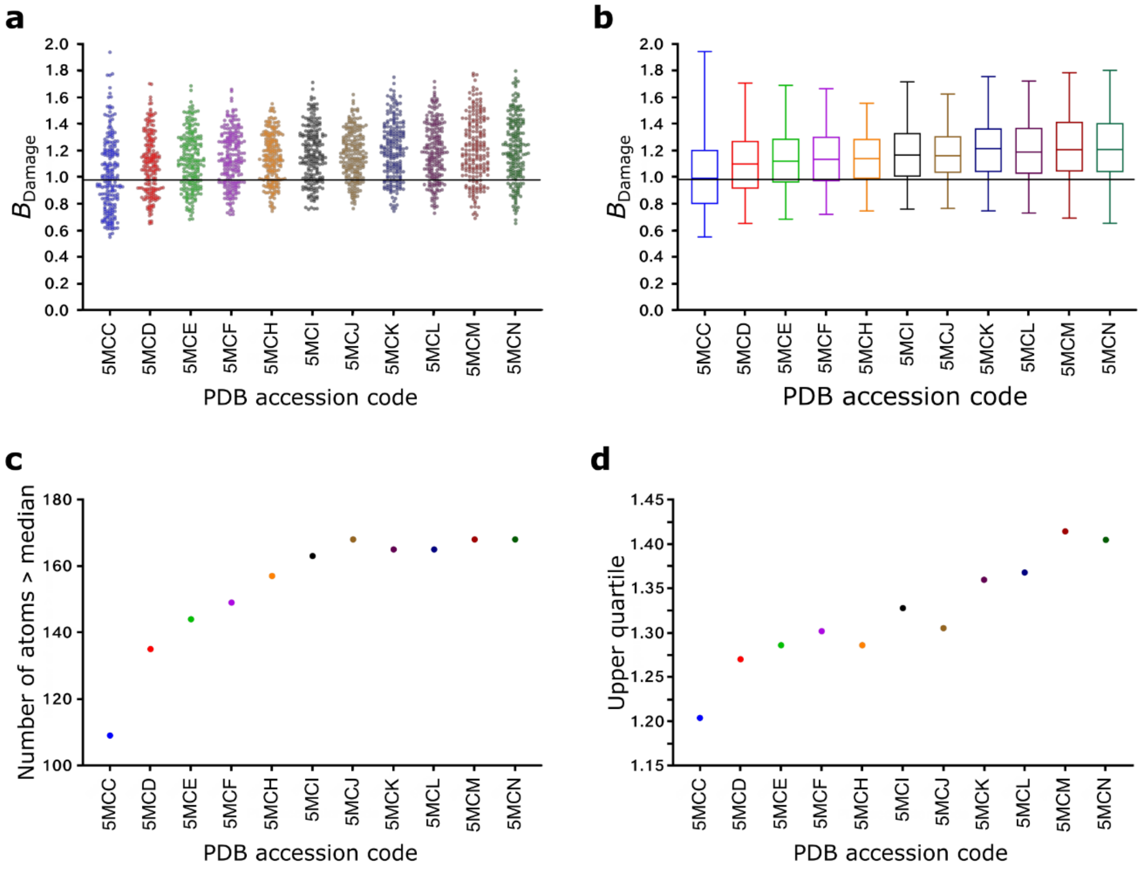

a Swarm plots and **b** box plots representing the distribution of the B_{Damage} values of the 204 glutamate and aspartate side chain oxygen atoms across the 11 increasingly damaged structures in the GH7 cellobiohydrolase damage series[1] (5MCC = lowest dose; 5MCN = highest dose). In **b**, boxes demarcate the median, lower and upper quartiles, whilst the tails demarcate the minimum and maximum B_{Damage} values. The median B_{Damage} value of all atoms in the structure varies between 0.96 and 0.98 (depending on the structure): the black line in plots **a** and **b** has been drawn at B_{Damage} = 0.98. **c** Whilst the increase in the number of glutamate and aspartate side chain carboxyl group oxygen atoms with a B_{Damage} value greater than the median B_{Damage} value of all atoms in the structure plateaus, **d** the raw B_{Damage} values of these atoms (represented here by the 75th percentile) continue to increase. Source data are provided as a Source Data file.

Supplementary Figure 3: Setting a lower threshold for the protein size amenable to B_{net} analysis.

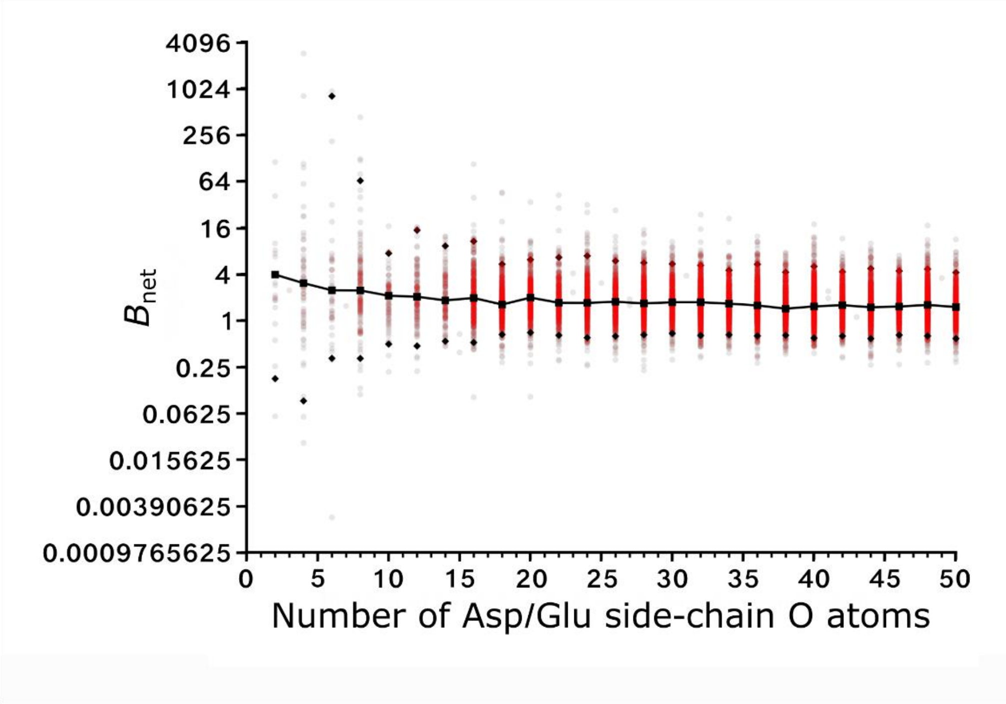

B_{net} vs. the number of aspartate/glutamate side chain oxygen atoms for structures containing up to 50 such atoms. We expect these two variables to be independent, and therefore for the range and distribution of B_{net} values to be the same regardless of the number of side-chain carboxyl group oxygen atoms. However, for very small structures this is not the case, hence we impose a threshold of a minimum 20 aspartate/glutamate side chain oxygen atoms in order to meet this assumption. Red circles represent B_{net} values of individual structures, whilst black squares and diamonds represent the median B_{net} value plus 95% confidence limits (namely the 2.5^{th} and 97.5^{th} percentiles), respectively, at every number of side-chain carboxyl group oxygen atoms for which there are 10 or more example structures in the dataset (of 93,978 PDB-REDO structures described in the main text). Note upper confidence limits are not plotted for structures with fewer than six side-chain carboxyl group oxygen atoms because their values are infinite. Source data are provided as a Source Data file.

Supplementary Figure 4: Damage to Asp/Glu in high B_{net} structures.

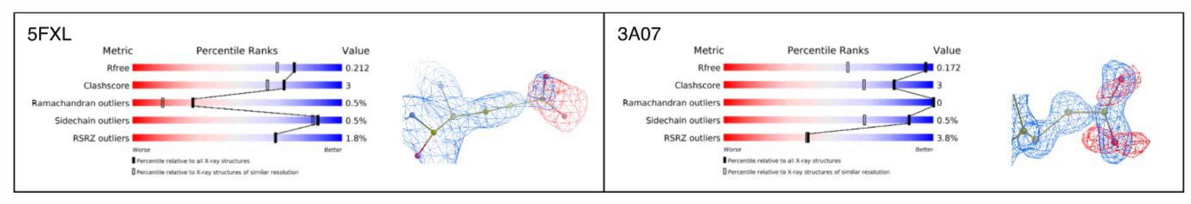

There is clear evidence of radiation damage to aspartate/glutamate side chain carboxyl groups in 5FXL[2] and 3A07[3] in their electron density maps. A representative damaged carboxyl group is shown for each structure. Damage to the disulfide bonds in these structures is shown in Figure 6. 2mF_{obs} – DF_{calc} maps (blue) are contoured at 1.5 rmsd; F_{obs} – F_{calc} difference density maps are contoured at +/- 3.0 rmsd (green/red).

Supplementary Figure 5: B_{net} and B_{net}-percentile as a function of PDB deposition year.

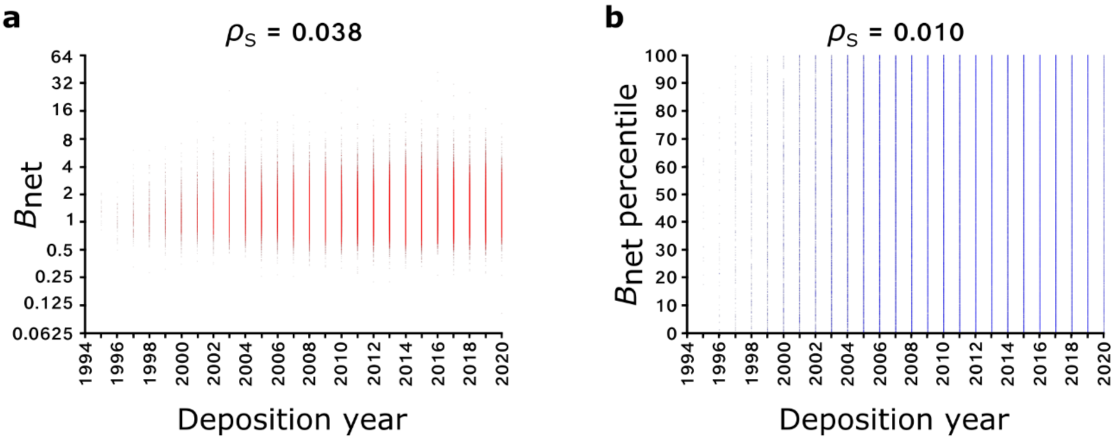

Scatter plots of **a** B_{net} and **b** B_{net}-percentile vs. deposition year for our dataset of 93,978 PDB-REDO structures. Spearman's rank correlation coefficients (ρ_S) indicate no correlation between these variables. Source data are provided at https://doi.org/10.5281/zenodo.5566558.

Supplementary Tables

Series	B_{net} (Asp/Glu)			B_{net} (Asn/Gln)			Wilson *B*-factor		
	Gradient (MGy^{-1})	y-intercept	R^2	Gradient (MGy^{-1})	y-intercept	R^2	Gradient (Å^2/MGy)	y-intercept (Å^2)	R^2
Serrano-Posada *et al.*, 2015 [4]	0.0532	2.32	0.00570	-0.124	2.85	0.0242	0.0558	11.5	0.00388
Ferraroni *et al.*, 2012 [5]	0.303	0.961	0.949	0.413	1.27	0.612	-1.58	21.2	0.665
Bui *et al.*, 2014, series 1[6]	0.313	1.31	0.761	-0.0200	1.88	0.988	0.922	10.8	0.998
Bui *et al.*, 2014, series 2[6]	0.534	1.84	1.00	-0.0696	2.36	0.764	0.376	9.88	0.998
Sutton *et al.*, 2013[7]	0.285	1.31	0.365	-0.0579	1.70	0.0235	0.261	10.7	0.703
Castellvi *et al.*, 2019[8]	1.35	1.68	0.868	-0.113	1.64	0.968	0.894	6.29	1.00
Zarate-Romero *et al.*, 2019[9]	0.284	1.24	0.994	0.00293	1.17	0.00419	-0.273	15.1	0.0716
Taberman *et al.*, 2019[10]	0.213	1.74	0.777	0.0417	1.18	0.263	0.546	10.1	0.984
Dubnovitsky *et al.*, 2005[11]	0.230	1.35	0.977	0.0271	2.55	0.104	0.602	14.8	0.991
Fioravanti *et al.*, 2007[12]	0.289	1.39	0.999	-0.0327	2.24	0.949	0.368	17.1	0.904
Juers & Weik, 2011[13]	0.303	1.51	0.843	0.0165	1.00	0.315	0.697	10.9	0.962
Correy, *et al.,* 2016[14]	0.212	1.36	0.911	0.00940	1.32	0.0543	0.995	34.4	0.869
Russi *et al.*, 2017, series 1[15]	0.308	1.56	0.598	-0.101	2.50	0.295	0.537	12.6	0.992
Russi *et al.*, 2017, series 2[15]	0.145	0.888	0.836	-0.0476	1.54	0.274	0.597	23.4	0.985
Polyakov *et al.*, 2019, series 1[16]	0.516	1.88	0.958	-0.0377	1.84	0.0822	0.427	9.66	0.750
Polyakov *et al.*, 2019, series 2[16]	0.186	2.12	0.613	0.0968	1.87	0.339	0.768	8.49	0.580
Polyakov *et al.*, 2019, series 3[16]	0.677	1.87	0.991	-0.0143	1.92	0.0663	0.936	8.77	0.912
De la Mora *et al.*, 2011[17]	0.165	1.34	0.838	-0.0119	1.02	0.839	0.442	13.6	0.997
Pechkova *et al.*, 2009, series 1 [18]	0.0173	1.76	0.924	-0.0174	4.95	0.689	0.0198	5.95	0.962
Pechkova *et al.*, 2009, series 2[18]	0.0452	2.20	0.491	-0.00914	3.83	0.257	0.204	9.36	0.774
Fukuda *et al.*, 2016 [19]	0.156	2.82	0.946	0.00539	1.56	0.0205	0.0888	9.84	0.602
Hasegawa *et al.*, 2017[20]	-0.0137	2.02	0.901	-0.0125	1.77	0.582	0.237	10.5	0.985
Bury *et al.*, 2017 *[1]	0.221	1.03	0.986	0.00303	1.10	0.0405	0.669	22.8	0.656

Supplementary Table 1: Gradients and y-intercepts of the lines of best fit calculated for the B_{net} vs. dose data for the 23 selected radiation damage series. All values rounded to 3sf. * symbol indicates final four structures have been excluded from the calculation of the line of best fit plotted for the series in Figure 3.

Metric	B_{net} (Asp/Glu)		B_{net} (Asn/Gln)		Wilson *B*-factor	
	Gradient (MGy^{-1})	y-intercept	Gradient (MGy^{-1})	y-intercept	Gradient ($Å^2$/MGy)	y-intercept ($Å^2$)
Mean average	0.291	1.65	-0.00237	1.96	0.351	13.6
Relative standard deviation	97.4%	27.2%	4360%	47.8%	154%	51.2%

Supplementary Table 2: Mean average and relative standard deviation scores calculated for the gradients and y-intercepts of the lines of best fit plotted between B_{net} and dose for the 23 radiation damage series listed in Supplementary Table 1. All values rounded to 3sf.

Publication	Protein	PDB accession codes	Doses (MGy)
Serrano-Posada *et al.*, 2015[4]	*Thermus thermophilus* HB27 multicopper oxidase	2YAE, 2YAF, 2YAH, 2YAM, 2YAO, 2YAP, 2YAQ, 2YAR	0.2, 0.4, 0.6, 0.8, 1.0, 1.2, 1.4, 1.6
Ferraroni *et al.*, 2012[5]	*Steccherinum ochraceum* blue laccase	3T6W, 3T6X, 3T6Z, 3T71	0.14, 0.28, 0.84, 1.26
Bui *et al.*, 2014 (series 1)[6]	*Aspergillus flavus* urate oxidase (with 5-PMUA)	4CW2, 4CW6, 4CW3	0.0025, 0.092, 0.665
Bui *et al.*, 2014 (series 2)[6]	*Aspergillus flavus* urate oxidase (with 5-PIU)	4D13, 4D17, 4D19	0.0022, 0.106, 1.75
Sutton *et al.*, 2013[7]	*Gallus gallus* lysozyme	4H8X, 4H8Y, 4H8Z, 4H90, 4H91, 4H92, 4H93, 4H94, 4H9A, 4H9B, 4H9C, 4H9E, 4H9F, 4H9H, 4H9I	0.07, 0.14, 0.21, 0.28, 0.35, 0.42, 0.49, 0.56, 0.63, 0.70, 0.77, 0.84, 0.91, 0.98, 1.05
Castellvi *et al.*, 2019[8]	*Homo sapiens* aldose reductase	6F7R, 6F81, 6F82	0.03, 0.75, 1.65
Zarate-Romero *et al.*, 2019[9]	*Neurospora crassa* catalase-3	6NSW, 6NSY, 6NSZ, 6NT0, 6NT1	0.135, 0.263, 0.526, 1.31, 2.89
Taberman *et al.*, 2019[10]	*Streptomyces rubiginosus* xylose isomerase	6QRR, 6QRS, 6QRT, 6QRU, 6QRV, 6QRW, 6QRX	0.13, 0.76, 1.38, 2.01, 2.63, 3.25, 3.88
Dubnovitsky *et al.*, 2005[11]	*Bacillus alcalophilus* phosphoserine aminotransferase	2BHX, 2BI1, 2BI2, 2BI3, 2BI5, 2BI9, 2BIA	0.022, 0.099, 0.22, 0.88, 1.5, 2.9, 4.7
Fioravanti *et al.*, 2007[12]	*Haloarcula marismortui* malate dehydrogenase	2J5K, 2J5Q, 2J5R	1.2, 4.6, 8.2
Juers & Weik, 2011[13]	*Bacillus thermoproteolyticus* thermolysin	3P7P, 3P7Q, 3P7R, 3P7S	0.1, 2.5, 4.9, 7.2
Correy *et al.*, 2016[14]	*Lucilia cuprina* αE7 carboxylesterase	4QWM, 4UBI, 4UBJ, 4UBK, 4UBM, 4UBL	1.85, 3.7, 5.55, 7.4, 9.26, 11.11
Russi *et al.*, 2017 (series 1)[15]	*Homo sapiens* cyclophilin A	5KUL, 5KUN, 5KUO, 5KUQ, 5KUR, 5KUS, 5KUU, 5KUV, 5KUW	1.11, 2.22, 3.33, 4.44, 5.55, 6.66, 7.77, 8.88, 9.99
Russi *et al.*, 2017 (series 2)[15]	*Thaumatococcus daniellii* thaumatin	5KVW, 5KVX, 5KVZ, 5KW0	1.86, 3.72, 5.58, 9.3
Polyakov *et al.*, 2019 (series 1)[16]	*Steccherinum murashkinskyi* laccase	6RGH, 6RGP, 6RHH, 6RHI, 6RHO	0.015, 0.165, 0.315, 1.215, 4.065
Polyakov *et al.*, 2019 (series 2)[16]	*Steccherinum murashkinskyi* laccase (with chloride)	6RHR, 6RHU, 6RHX, 6RI0, 6RI2	0.015, 0.165, 0.315, 1.215, 4.065

Polyakov *et al.*, 2019 (series 3)[16]	*Steccherinum murashkinskyi* laccase (with fluoride)	6RI4, 6RI6, 6RI8, 6RII, 6RIK	0.013, 0.4, 0.8, 1.2, 5.2
De la Mora *et al.*, 2011[17]	*Gallus gallus* lysozyme	2YBH, 2YBI, 2YBJ, 2YBL, 2YBM, 2YBN	2.31, 6.62, 12.3, 17.9, 23.3, 28.6
Pechkova *et al.*, 2009 (series 1)[18]	*Tritirachium album* proteinase K, LB nanotemplate	3DDZ, 3DE0, 3DE1, 3DE2	9.6, 22.45, 31.2, 44.05
Pechkova *et al.*, 2009 (series 2)[18]	*Tritirachium album* proteinase K, classical hanging drop	3DE3, 3DE4, 3DE5, 3DE6, 3DE7	0.85, 9.6, 22.45, 31.2, 44.05
Fukuda *et al.*, 2016[19]	*Geobacillus thermodenitrificans* copper nitrite reductase	4YSO, 4YSP, 4YSQ, 4YSR, 4YSS, 4YST, 4YSU	0.064, 8.316, 8.38, 16.632, 16.696, 24.948, 25.012
Hasegawa *et al.*, 2017[20]	*Photinus pyralis* luciferin-regenerating enzyme	5GX1, 5GX2, 5GX3, 5GX4, 5GX5	1.1, 3.3, 6.6, 13.2, 25.3
Bury *et al.*, 2017[1]	*Daphnia pulex* GH7 family cellobiohydrolase	5MCC, 5MCD, 5MCE, 5MCF, 5MCH, 5MCI, 5MCJ, 5MCK, 5MCL, 5MCM, 5MCN	1.11, 3.27, 5.43, 7.59, 9.75, 11.9, 14.1, 16.2, 18.4, 20.6, 22.7

Supplementary Table 3: PDB accession codes and associated doses of the PX structures in the 23 radiation damage series analysed. All doses were calculated with RADDOSE (versions 1[21], 2[22] or 3D[23]). Full metadata collated for each structure in the series is available at https://doi.org/10.5281/zenodo.5566557.

Series	Gradient of line of best fit plotted between B_{net} and dose (MGy^{-1}) (3sf)		
	Original dataset from PDB	Dataset from PDB-REDO	Original dataset subjected to unrestrained *B*-factor refinement
Serrano-Posada *et al.*, 2015[4]	0.0532	0.142	-0.0657
Pechkova *et al.*, 2009, series 1[18]	0.0173	-0.0163	-0.000767
Pechkova *et al.*, 2009, series 2[18]	0.0452	0.0222	0.0543
Hasegawa *et al.*, 2017[20]	-0.0137	0.105	0.127

Supplementary Table 4: Further exploration of the relationship between B_{net} and dose for four radiation damage datasets (a subset of the 23 datasets listed in Supplementary Tables 1 and 3), each of whose calculated line of best fit between B_{net} and dose has an unusually low gradient. These gradients, calculated for structures downloaded from the PDB, are compared with the gradients of the lines of best fit measured between B_{net} and dose for: i) equivalent structures downloaded from the PDB-REDO databank; and ii) the original (*i.e.* PDB) structures subjected to three macrocycles of unrestrained *B*-factor refinement (see Methods for further details).

Structure property	B_{net} ρ_S (2sf)	B_{net}-percentile ρ_S (2sf)
Resolution (Å)	-0.42	0.0078
R_{work}	-0.41	-0.15
R_{free}	-0.40	-0.12
Temperature (K)	-0.016	-0.020
Molecular mass (kDa)	-0.18	-0.0082
Number of Asp/Glu side chain oxygen atoms	-0.20	-0.032
% Asp/Glu	-0.091	-0.067
B-factor restraint weight	-0.12	-0.17

Supplementary Table 5: Spearman's rank correlation coefficients (ρ_S) of the B_{net} and B_{net}-percentile metrics with eight variables identified as possibly influencing their values (resolution, R_{work}, R_{free}, temperature, molecular mass, the number of aspartate/glutamate side chain oxygen atoms, the percentage of aspartate and glutamate residues in the structure, and *B*-factor restraint weight).

PDB accession code	B_{net} (PDB-REDO structure)	B_{net} percentile (PDB-REDO structure) (rounded to 4sf if less than 1)	Resolution (3sf)	Resolution bin (min – max) (3sf)	B_{net} (PDB structure)
5WUC[24]	42.0	1	1.60	1.60 – 1.60	18.6
5FXL[2]	34.1	1	1.78	1.77 – 1.79	29.6
5XQP[25]	31.4	1	1.00	0.900 – 1.10	12.9
3S8S	28.5	1	1.30	1.28 – 1.32	16.3
3UX1[26]	27.5	1	2.80	2.80 – 2.80	25.1
1V70	26.6	0.9991	1.30	1.28 – 1.32	12.1
6Q5R[27]	25.4	0.9996	1.61	1.60 – 1.62	48.5
3A07[3]	25.1	1	1.19	1.15 – 1.23	10.1
6BKL[28]	23.4	1	2.00	2.00 – 2.00	10.4
2XMK[29]	20.9	1	1.35	1.33 – 1.37	28.1

Supplementary Table 6: The B_{net}, B_{net}-percentile and resolution values, plus the resolution bin considered when calculating B_{net}-percentile scores, for the 10 structures with the highest B_{net} values in the PDB-REDO databank as of 19th November 2020. For comparison, the B_{net} values of the original structures deposited by the authors in the PDB are also provided.

Supplementary References

1. Bury, C.S., Carmichael, I. & Garman, E.F. OH cleavage from tyrosine: debunking a myth. *J. Syn. Rad.* **24**, 7-18 (2017).
2. Hirano, Y., Amano, Y., Yonemura, S. & Hakoshima, T. The force-sensing device region of alpha-catenin is an intrinsically disordered segment in the absence of intramolecular stabilization of the autoinhibitory form. *Genes Cells* **23**, 370-385 (2018).
3. Tanaka, H. et al. Mechanism by which the lectin actinohivin blocks HIV infection of target cells. *Proc. Natl Acad. Sci. USA* **106**, 15633-15638 (2009).
4. Serrano-Posada, H. et al. X-ray-induced catalytic active-site reduction of a multicopper oxidase: Structural insights into the proton-relay mechanism and O2-reduction states. *Acta Cryst. D.* **71**, 2396-2411 (2015).

5. Ferraroni, M. et al. Reaction intermediates and redox state changes in a blue laccase from Steccherinum ochraceum observed by crystallographic high/low X-ray dose experiments. *J. Inorg. Biochem.* **111**, 203-209 (2012).
6. Bui, S. et al. Direct evidence for a peroxide intermediate and a reactive enzyme-substrate-dioxygen configuration in a cofactor-free oxidase. *Angew Chem. Int. Ed. Engl.* **53**, 13710-4 (2014).
7. Sutton, K.A. et al. Insights into the mechanism of X-ray-induced disulfide-bond cleavage in lysozyme crystals based on EPR, optical absorption and X-ray diffraction studies. *Acta Cryst. D.* **69**, 2381-94 (2013).
8. Castellvi, A. et al. Efficacy of aldose reductase inhibitors is affected by oxidative stress induced under X-ray irradiation. *Sci. Rep.* **9**, 3177-3177 (2019).
9. Zarate-Romero, A., Stojanoff, V., Cohen, A.E., Hansberg, W. & Rudino-Pinera, E. X-ray driven reduction of Cpd I of Catalase-3 from N. crassa reveals differential sensitivity of active sites and formation of ferrous state. *Arch. Biochem. Biophys.* **666**, 107-115 (2019).
10. Taberman, H., Bury, C.S., van der Woerd, M.J., Snell, E.H. & Garman, E.F. Structural knowledge or X-ray damage? A case study on xylose isomerase illustrating both. *J. Syn. Rad.* **26**, 931-944 (2019).
11. Dubnovitsky, A.P., Ravelli, R.B., Popov, A.N. & Papageorgiou, A.C. Strain relief at the active site of phosphoserine aminotransferase induced by radiation damage. *Protein Sci* **14**, 1498-507. (2005).
12. Fioravanti, E., Vellieux, F.M., Amara, P., Madern, D. & Weik, M. Specific radiation damage to acidic residues and its relation to their chemical and structural environment. *J. Syn. Rad.* **14**, 84-91. (2007).
13. Juers, D.H. & Weik, M. Similarities and differences in radiation damage at 100 K versus 160 K in a crystal of thermolysin. *J. Syn. Rad.* **18**, 329-337 (2011).
14. Correy, G.J. et al. Mapping the Accessible Conformational Landscape of an Insect Carboxylesterase Using Conformational Ensemble Analysis and Kinetic Crystallography. *Structure* **24**, 977-987 (2016).
15. Russi, S. et al. Conformational variation of proteins at room temperature is not dominated by radiation damage. *J. Syn. Rad.* **24**, 73-82 (2017).
16. Polyakov, K.M., Gavryushov, S., Fedorova, T.V., Glazunova, O.A. & Popov, A.N. The subatomic resolution study of laccase inhibition by chloride and fluoride anions using single-crystal serial crystallography: insights into the enzymatic reaction mechanism. *Acta Cryst. D.* **75**, 804-816 (2019).
17. De la Mora, E., Carmichael, I. & Garman, E.F. Effective scavenging at cryotemperatures: further increasing the dose tolerance of protein crystals. *J. Syn. Rad.* **18**, 346-57. (2011).
18. Pechkova, E., Tripathi, S., Ravelli, R.B., McSweeney, S. & Nicolini, C. Radiation stability of proteinase K crystals grown by LB nanotemplate method. *J. Struct. Biol.* **168**, 409-418 (2009).
19. Fukuda, Y. et al. Redox-coupled structural changes in nitrite reductase revealed by serial femtosecond and microfocus crystallography. *J. Biochem.* **159**, 527-538 (2016).
20. Hasegawa, K. et al. Development of a dose-limiting data collection strategy for serial synchrotron rotation crystallography. *J. Syn. Rad.* **24**, 29-41 (2017).
21. Murray, J.W., Garman, E.F. & Ravelli, R.B.G. X-ray absorption by macromolecular crystals: the effects of wavelength and crystal composition on absorbed dose. *J. Appl. Cryst.* **37**, 513-522 (2004).

22. Paithankar, K.S., Owen, R.L. & Garman, E.F. Absorbed dose calculations for macromolecular crystals: improvements to RADDOSE. *J. Syn. Rad.* **16**, 152-162 (2009).
23. Zeldin, O.B., Gerstel, M. & Garman, E.F. RADDOSE-3D: time- and space-resolved modelling of dose in macromolecular crystallography. *J. Appl. Cryst.* **46**, 1225-1230 (2013).
24. Su, M. et al. Structural basis for conductance through TRIC cation channels. *Nat. Commun.* **8** 15103-15103 (2017).
25. Mahatabuddin, S. et al. Polypentagonal ice-like water networks emerge solely in an activity-improved variant of ice-binding protein. *Proc. Natl Acad. Sci. USA* **115** 5456-5461 (2018).
26. Dimattia, M.A. et al. Structural insight into the unique properties of adeno-associated virus serotype 9. *J. Virol.* **86**, 6947-6958 (2012).
27. Rhys, G.G. et al. Navigating the Structural Landscape of De Novo alpha-Helical Bundles. *J. Am. Chem. Soc.* **141**, 8787-8797 (2019).
28. Thomaston, J.L., Polizzi, N.F., Konstantinidi, A., Wang, J., Kolocouris, A., DeGrado, W.F. Inhibitors of the M2 Proton Channel Engage and Disrupt Transmembrane Networks of Hydrogen-Bonded Waters. *J. Am. Chem. Soc.* **140**, 15219-15226 (2018).
29. Badarau, A., Firbank, S.J., Mccarthy, A.A., Banfield, M.J. & Dennison, C. Visualizing the Metal-Binding Versatility of Copper Trafficking Sites. *Biochemistry* **49**, 7798-7810 (2010).
